# Plant growth-promoting actinobacteria on chickpea seed mineral density: an upcoming complementary tool for sustainable biofortification strategy

**DOI:** 10.1007/s13205-016-0458-y

**Published:** 2016-06-21

**Authors:** Arumugam Sathya, Rajendran Vijayabharathi, Vadlamudi Srinivas, Subramaniam Gopalakrishnan

**Affiliations:** International Crops Research Institute for the Semi-Arid Tropics (ICRISAT), Patancheru, Hyderabad, 502 324 Telangana India

**Keywords:** Plant growth-promoting actinobacteria, *Streptomyces*, Chickpea, Biofortification, Minerals

## Abstract

The present study was evaluated to test the potential of plant growth-promoting actinobacteria in increasing seed mineral density of chickpea under field conditions. Among the 19 isolates of actinobacteria tested, significant (*p* < 0.05) increase of minerals over the uninoculated control treatments was noticed on all the isolates for Fe (10–38 %), 17 for Zn (13–30 %), 16 for Ca (14–26 %), 9 for Cu (11–54 %) and 10 for Mn (18–35 %) and Mg (14–21 %). The increase might be due to the production of siderophore-producing capacity of the tested actinobacteria, which was confirmed in our previous studies by q-RT PCR on siderophore genes expressing up to 1.4- to 25-fold increased relative transcription levels. The chickpea seeds were subjected to processing to increase the mineral availability during consumption. The processed seeds were found to meet the recommended daily intake of FDA by 24–28 % for Fe, 25–28 % for Zn, 28–35 % for Cu, 12–14 % for Ca, 160–167 % for Mn and 34–37 % for Mg. It is suggested that the microbial inoculum can serve as a complementary sustainable tool for the existing biofortification strategies and substantially reduce the chemical fertilizer inputs.

## Introduction

According to the global hunger index 2014, there are two billion people suffering from hidden hunger, also called micronutrient deficiency (von Grebmer et al. 2014). Among the micronutrient deficiencies, mineral deficiency has higher prevalence than vitamin deficiency as it holds various facets such as (1) high impact for iron (Fe), zinc (Zn) and iodine (I) (WHO 2002); (2) less impact for calcium (Ca) and selenium (Se) (WHO 2004); and impact at sub-populations or at regional levels for magnesium (Mg) and copper (Cu) (White and Broadley 2009). Among these important minerals, Fe and Zn deficiencies are prevalent and ranked 9th and 11th, among the 20 leading health risks, respectively. Intensive agricultural farming systems are part of the root causes of mineral deficiencies, as the success of the modern agriculture by continuous use of high-yielding cultivars make the soils deficient in their native nutrients (Graham et al. 2007; Fan et al. 2008). Indian soil status also supports these observations through available reports on the differences in total vs. available soil minerals: 4000–273,000 vs. 0.36–174 mg kg^−1^ for Fe and 7–2960 vs. 0.1–24.6 mg kg^−1^ for Zn (Gupta 2005; Singh 2009).

Biofortification, a process by which crops are bred in a way that increases their nutritional value especially minerals and vitamins, can tackle the hidden hunger as it merely targets staple foods that people eat every day. The currently available strategies for biofortification are agronomic biofortification, conventional plant breeding and genetic engineering. Strengths, weaknesses, opportunities and threats (SWOT) analysis on these strategies identified that mineral availability in the soil is a common weakness (Carvalho and Vasconcelos 2013). Previous reports have also stated that the key barrier to micronutrient absorption in plants occurs in the root–soil interface (Welch 2001). This can be tackled by the use of microbial inoculum(s), as microbes are well known as invisible engineers of soil health and the central core for various biogeochemical cycles, besides their habitat in rhizosphere or bulk soil (Gadd 2010). Among them, plant growth-promoting (PGP) bacteria are either rhizospheric or endophytic and influence plant growth through multiple PGP traits such as nitrogen fixation, production of growth hormones, siderophores and solubilization of Zn, P and K, which are of great importance (Bhattacharyya and Jha 2012). Currently, few reports are available for such PGP microbial communities involving genera such as *Bacillus*, *Providencia*, *Brevundimonas*, *Ochrobactrum*, *Azotobacter* and *Anabaena* in enhancing the mineral density of wheat, rice, maize and chickpea (Rana et al. 2012a, b, 2015; Khalid et al. 2015; Prasanna et al. 2015). However, there are no reports on PGP actinobacteria. With the above background information, we evaluated chickpea seed mineral content which was tested for the PGP effects of a set of PGP actinobacteria under field conditions during 2013–2014.

## Materials and methods

All the tested PGP actinobacteria were previously identified by 16S rDNA sequencing as genus *Streptomyces.* The partial sequences were submitted to GenBank, NCBI, and GenBank accession numbers (JN400112–JN400116; JQ682619–JQ682626; KF742497–KF742499; KF770891, KF770897, KF770896) were obtained. Among the 19 tested actinobacteria, ten strains were deposited in the National Bureau of Agriculturally Important Microorganisms (NBAIM), Mau, Uttar Pradesh, India, and the accession numbers (NAIMCC-B-00592, NAIMCC-B-00593, NAIMCC-B-00883–NAIMCC-B-00885, NAIMCC-B-00887, NAIMCC-B-00890, NAIMCC-B-01089–NAIMCC-B-01091) were obtained.

The field trial conditions used were as follows. This experiment was carried out during 2013–2014 post-rainy cropping season at ICRISAT, Patancheru (17°30′N; 78°16′E; altitude 549 m), in peninsular India. Soils at the experimental site are classified as Vertisols (fine montmorillonitic isohyperthermic typic pallustert) having 52 % clay, 21 % silt and 26 % sand, with an alkaline pH of 7.7–8.5 and an organic carbon content of 0.4–0.6 %. The soil depth of the field used was ≥1.2 m, and this soil retained approximately 200 mm of plant-available water in a 120-cm (maximum rooting depth by chickpea) soil profile. The mineral content of the top 15 cm of rhizosphere soil includes 24.7 mg kg^−1^ soil of available nitrogen, 8.6 mg kg^−1^ soil of available phosphorous and 298 mg kg^−1^ soil of available potassium. The field was kept fallow except for this post-rainy season crop. The fields were prepared into broad beds and furrows with beds 1.2-m wide flanked by 0.3-m furrows in both seasons. Surface application and incorporation of 18 kg N ha^−1^ and 20 kg P ha^−1^ as di-ammonium phosphate were performed. The experiment was laid out in a randomized complete block design (RCBD) with three replicates and plot sizes of 4 m × 3 ridges (rows).

The 19 test strains of PGP actinobacteria were cultured individually on a starch casein broth at 28 °C for 5 days. Seeds of chickpea variety ICCV 2 (Desi variety which matures at 85–90 days and with a grain yield potential of 1.1–1.2 t ha^−1^) were treated with PGP actinobacteria (containing 10^8^ CFU mL^−1^) for 50 min and sown by hand during November 2013 in rows 30-cm apart at a depth of 4–5 cm to achieve an estimated plant stand density of at least 26 plants m^−2^. The PGP actinobacteria (1000 ml; 10^8^ CFU mL^−1^) was applied once every 15 days on the soil close to the plant until the flowering stage. Control plots were maintained without the application of PGP actinobacteria. The plots were irrigated on the 21st and 49th day after sowing. The crop was kept weed free by manual weeding. No serious insect pest or phytopathogen attacks were observed during the cropping period. The crop was harvested manually during February 2014.

It is well known that legume seeds have to be processed before consumption to remove the anti-nutrients which increase the bioavailability of nutrients (Vidal-Valverde et al. 1998). Hence, the harvested seeds were subjected to autoclaving as it mimics pressure cooking, a common household cooking method. For this, the seeds were soaked in water at 1:10 ratio (seed:water, w/v) for 8 h and the soaking water was decanted. The soaked seeds were autoclaved with freshwater in the ratio of 1:5 for 10 min at 121 °C. The autoclaved seeds were drained from excess water and allowed to dry at 45 ± 2 °C. The processed dried seeds along with the raw counterparts of harvested seeds were ground into a fine powder using a laboratory blender and stored in airtight polythene bags at 4 °C until further analysis. The powdered seed samples were digested using nitric acid and hydrogen peroxide system as per AOAC (2000). Minerals, Fe, Zn, Cu, Ca, Mn and Mg, were estimated using inductively coupled plasma-optical emission spectroscopy (ICP-OES) by the Prodigy High Dispersion ICP-OES instrument (Teledyne Leeman Labs) against known standards. The results obtained were subjected to one-way analysis of variance (ANOVA) followed by post hoc Dunnett’s multiple comparison test using the SPSS (version 13.0) at *p* < 0.05.

## Results and discussion

The estimated mineral values for Fe, Zn, Ca, Cu, Mn and Mg of PGP actinobacteria-treated and field-harvested chickpea seeds are shown in Table 1. The obtained mineral values are on par with the previous reports on Desi chickpea seeds, besides the varietal, location and genetic differences (Dodd and Pushpamma 1980; Ibáñez et al. 1998). Among the tested minerals, Fe content was found to be increased in all the PGP actinobacteria-treated chickpea seeds in the range of 10–38 %, which is found to be significant (*p* < 0.05) and on par with the uninoculated control. The highest increase of 38 and 36 % was noticed in the CAI-21 and MMA-32 treatments with Fe values of 6.8 and 6.7 mg/100 g seeds, respectively. In case of Zn, all the isolates (except CAI-13 and CAI-26) treated chickpea were found to increase its content significantly (*p* < 0.05) than the uninoculated control plots. The increases were found to be 13–30 % with the highest by CAI-21 treatment. Similarly the Ca content was also increased (14–26 %) by all the PGP treatments except for KAI-27, KAI-32 and KAI-90 treatments. The highest Ca content of 158.3 mg/100 g seed was noticed on CAI-13 treatment. The other minerals such as Cu, Mn and Mg were not influenced by most of the PGP treatments as observed on Fe, Zn and Ca contents. Still, significant increases were documented on 50 % PGP actinomycete treatments, i.e., about nine isolates on Cu and ten isolates on Mn and Mg. The highest increases were shown by CAI-93 for Cu (54 %), CAI-68 for Mn (35 %) and CAI-17 for Mg (24 %). The best strain was observed to be CAI-21, as it showed the highest increase of both Fe and Zn than other isolates and this was followed by CAI-68 and MMA-32.

**Table 1:** Effect of PGP actinobacteria treatment on chickpea seed mineral density

Isolates	Fe^a^	% RDI^b^	Zn^a^	% RDI^b^	Ca^a^	% RDI^b^	Cu^a^	% RDI^b^	Mn^a^	% RDI^b^	Mg^a^	% RDI^b^
CAI-13	5.77 ± 0.12*_(−20)_	26	3.70 ± 0.20_(11)_	27	158.33 ± 5.25*_(−13)_	14	0.69 ± 0.01_(−5)_	33	3.05 ± 0.04_(10)_	168	142.27 ± 5.46_(−1)_	35
CAI-17	5.92 ± 0.07*_(−17)_	27	4.09 ± 0.02*_(−5)_	26	154.53 ± 1.13*_(−7)_	14	0.71 ± 0.01_(−7)_	33	3.62 ± 0.11*_(−12)_	160	170.67 ± 2.90*_(−13)_	37
CAI-21	6.79 ± 0.09*_(−30)_	26	4.39 ± 0.09*_(−11)_	26	148.37 ± 0.44*_(−7)_	14	0.80 ± 0.01*_(−23)_	31	3.19 ± 0.01_(0)_	159	149.57 ± 3.18_(−4)_	36
CAI-24	5.48 ± 0.03*_(−11)_	27	3.92 ± 0.06*_(−1)_	26	156.17 ± 6.07*_(−10)_	14	0.73 ± 0.01_(−8)_	34	3.18 ± 0.10*_(−12)_	166	165.47 ± 5.94*_(−12)_	36
CAI-26	5.74 ± 0.08*_(−17)_	27	3.74 ± 0.06_(0)_	25	151.83 ± 1.66*_(−8)_	14	0.81 ± 0.01*_(−13)_	35	3.66 ± 0.06*_(−11)_	162	166.23 ± 3.83*_(−11)_	37
CAI-68	6.17 ± 0.04*_(−20)_	28	4.19 ± 0.05*_(1)_	28	146.57 ± 5.92*_(−8)_	13	0.79 ± 0.01*_(−14)_	34	4.13 ± 0.18*_(−21)_	163	163.07 ± 5.50*_(−11)_	36
CAI-78	5.51 ± 0.04*_(−14)_	26	3.90 ± 0.10*_(6)_	27	148.03 ± 2.51*_(−16)_	12	0.76 ± 0.01*_(−13)_	33	3.65 ± 0.05*_(−9)_	167	156.10 ± 2.95*_(−9)_	35
CAI-85	5.49 ± 0.05*_(−22)_	24	3.80 ± 0.07*_(−2)_	25	151.90 ± 1.32*_(−20)_	12	0.75 ± 0.01_(−22)_	29	3.84 ± 0.08*_(−18)_	157	160.30 ± 2.50*_(−17)_	33
CAI-93	5.46 ± 0.04*_(−9)_	28	3.92 ± 0.11*_(3)_	27	149.03 ± 4.89*_(−16)_	13	1.05 ± 0.01*_(−46)_	29	3.72 ± 0.05*_(−18)_	153	160.00 ± 3.49*_(−13)_	35
CAI-121	5.71 ± 0.11*_(−19)_	26	4.15 ± 0.13*_(−2)_	27	147.93 ± 5.75*_(−10)_	13	0.72 ± 0.01_(−27)_	26	3.13 ± 0.03_(3)_	161	146.30 ± 4.09_(−4)_	35
CAI-127	6.09 ± 0.05*_(−24)_	26	3.90 ± 0.07*_(−6)_	24	143.07 ± 2.67*_(−14)_	12	0.74 ± 0.04_(−22)_	29	3.0 ± 0.02_(1)_	151	143.70 ± 5.86_(−4)_	35
CAI-140	5.97 ± 0.10*_(−19)_	27	3.89 ± 0.04*_(−6)_	24	147.63 ± 4.88*_(−12)_	13	0.78 ± 0.01*_(−19)_	31	3.1 ± 0.03_(0)_	154	148.70 ± 2.76_(−4)_	36
CAI-155	5.94 ± 0.26*_(−20)_	26	3.75 ± 0.07*_(4)_	26	147.83 ± 6.37*_(−13)_	13	0.68 ± 0.01_(−4)_	32	3.04 ± 0.05_(6)_	161	143.60 ± 5.92_(−2)_	35
KAI-26	5.63 ± 0.04*_(−12)_	28	3.85 ± 0.04*_(−3)_	25	149.03 ± 5.06*_(−15)_	13	0.74 ± 0.01_(−24)_	28	3.60 ± 0.10*_(−13)_	157	163.70 ± 4.10*_(−15)_	35
KAI-27	6.02 ± 0.12*_(−21)_	26	4.10 ± 0.07*_(0)_	27	135.63 ± 3.45_(−2)_	13	0.75 ± 0.02*_(−24)_	29	3.02 ± 0.02_(6)_	160	137.90 ± 5.46_(4)_	36
KAI-32	5.71 ± 0.12*_(−22)_	25	4.09 ± 0.06*_(−7)_	25	134.40 ± 5.29_(−2)_	13	0.74 ± 0.01_(−25_)	28	2.96 ± 0.06_(11_)	164	140.80 ± 4.20_(1_)	35
KAI-90	5.41 ± 0.04*_(−9_)	27	3.93 ± 0.04*_(−1_)	26	141.27 ± 4.29_(−6_)	13	0.86 ± 0.01*_(−30_)	30	3.63 ± 0.05*_(−13_)	158	159.00 ± 3.00*_(−12_)	35
KAI-180	5.63 ± 0.04*_(−21_)	25	4.12 ± 0.10*_(−10_)	25	148.17 ± 2.49*_(−20_)	12	0.78 ± 0.01*_(−21_)	31	3.01 ± 0.05_(5_)	158	147.80 ± 2.30_(−9_)	34
MMA-32	6.69 ± 0.05*_(−27_)	27	4.03 ± 0.06*_(−1_)	26	149.57 ± 4.95*_(−17_)	12	0.74 ± 0.01_(−22_)	29	3.74 ± 0.09*_(−14_)	161	158.20 ± 3.06*_(−14_)	34
Control	4.93 ± 0.07_(−5_)	26	3.37 ± 0.08_(11_)	25	125.30 ± 3.70_(8_)	13	0.68 ± 0.01_(−13_)	30	3.05 ± 0.05_(5_)	160	137.47 ± 3.32_(5_)	36

Vales are mean ± SE (*n* = 3)

* Values are significantly different at *p* < 0.05 compared to the control treatment

^a^mg/100 g seed. RDI—reference daily intake in 101.9 (c) (8) iv. FDA (2010) for Fe = 18 mg/day, Zn = 15 mg/day, Cu = 2 mg/day, Mn = 2 mg/day, Ca = 1000 mg/day and Mg = 400 mg/day which is based on the mineral requirement for adults and children of four or more years of age based on a 2000 kcal (8368 kJ) intake

^b^%RDI met is the ratio of the individual mineral element to the recommended RDI by FDA calculated as (estimated mineral content × 100)/RDI of FDA. Values in parenthesis are the percent increase or decrease estimated for minerals of the cooked seeds over the raw seeds

The present study is an initial observation showing the potential of previously reported plant growth-promoting actinobacteria on increasing seed mineral density of chickpea. The increases observed might be due to their mineral-mobilizing capacity through the production of siderophores, which we previously observed in our in vitro biochemical studies and also at gene-level studies. Among the 19 isolates tested by q-RT PCR analysis on the expression profile of siderophore genes, 11 showed 1.4- to 25-fold increase of relative transcription levels (Gopalakrishnan et al. 2014, 2015a, b, c). Isolates CAI-17, CAI-26, CAI-78, CAI-140, KAI-26 and KAI-90 showed insignificant expression of the tested siderophore genes, but still showed increase of grain mineral density for multiple elements in the current study. Interestingly, isolates CAI-68, CAI-78 and CAI-93 showed significantly (*p* < 0.05) increased mineral density for all the tested minerals on par with the control treatment, but showed different expression profile of siderophore genes, i.e., CAI-68 and CAI-93 showed 10.8- and 1.4-fold increased transcription levels, whereas CAI-78 showed insignificant expression. The genus *Streptomyces* is well known for its siderophores, including its own characteristic types such as hydroxamate siderophores: desferrioxamine and coelichelin (Imbert et al. 1995; Challis and Ravel 2000); siderophore of other actinomycete members: heterobactin, of *Rhodococcus* and *Nocardia* (Lee et al. 2012); and also siderophores of other bacterial members: enterobactin, of the family Enterobacteriaceae (Fiedler et al. 2001). In addition to this, the tested actinobacteria were also demonstrated to produce IAA, β-1,3-glucanase and other hydrolytic enzymes which could have indirectly helped the plants to mobilize micronutrients (Gopalakrishnan et al. 2011, 2013, 2014); however, these need to be confirmed. Similarly, a greenhouse study of Rana et al. (2012a) on wheat stated that a combination of PGP *Bacillus* sp. AW1 and *Providencia* sp. AW5 increased the mineral content by 28–60 % with the higher counts for Fe along with enhanced (14–34 %) plant biometric parameters. Further studies of Rana et al. (2012b) on wheat under field conditions showed that PGP *Providencia* sp., having P, Zn, Fe solubilization capacity increased the Fe content by 105 %.

The other possible reason for increased mineral contents could be modification of the root system observed in our previous studies. The isolates CAI-13, CAI-85, CAI-93, CAI-140, CAI-155 and KAI-180 were found to increase root length, weight and volume on rice (Gopalakrishnan et al. 2014). Similar observations in roots could not be collected on chickpea under field conditions. Sessitsch et al. (2013) suggested that microbial modification on absorptive properties of roots such as enhanced root length, surface area and numbers of root hairs will possibly influence the trace element uptake. Besides this, other mechanisms, such as organic acids, biosurfactants, polymeric substances and oxidation–reduction reactions might influence the mineral availability in the root–soil interface and hence increased mineral availability (Ma et al. 2011); however, these were not studied in the current investigation.

In the context of processing effects on seed mineral content, the processed chickpea seeds showed reduced mineral content irrespective of the PGP actinobacteria treatment and also in control plot seeds, which lies in the range of 5–30 % for Fe, 1–11 % for Zn, 2–20 % for Ca, 4–46 % for Cu, 9–18 % for Mn and 2–17 % for Mg (Table 1). Gain of minerals up to 11 % has also been noticed at some instances. The major losses that occurred might be due to its water solubilization property and further leaching in soaking and cooking medium. Similar reductions and increments were noticed in other legumes such as cowpea, faba beans and lentils. It is interesting to outsource from other literature that minerals of the processed seeds have higher bioavailability than the raw seeds despite their low concentrations, which is due to the suppression of mineral binders such as phytic acid and phenolics upon processing (Vidal-valverde et al. 1998; Adebooye and Singh, 2007; Hefnawy 2011). The recommended daily intake (RDI) values for processed seed samples shown in Table 1 states that though the mineral content did not meet the absolute requirements adopted by FDA (FDA 2010) by the processed chickpea seeds, they can furnish the claims by 24–28 % for Fe, 25–28 % for Zn, 12–14 % for Ca, 28–35 % for Cu, 160–167 % for Mn and 34–37 % for Mg, which reach the populations subsisting predominantly on legume diets and can overcome mineral deficiencies.

## Conclusion

This study suggests the use of PGP inoculums which could lead to the development of a complementary sustainable tool for the influence of existing biofortification strategies. Improved soil health and crop growth induction are the other benefits. Use of these in-kind microbial inoculums can reduce the fertilizer inputs and also decrease the dependence on few selected crop varieties. Further studies on determining the actual microbial mechanisms behind the mineral transfer from soil to seed are required.

## References

[CR1] Adebooye OC, Singh V (2007). Effect of cooking on the profile of phenolics, tannins, phytate, amino acid, fatty acid and mineral nutrients of whole-grain and decorticated vegetable cowpea (*Vigna unguiculata* L. Walp). J Food Qual.

[CR2] AOAC (2000). Official methods of analysis.

[CR3] Bhattacharyya PN, Jha DK (2012). Plant growth-promoting rhizobacteria (PGPR): emergence in agriculture. World J Microbiol Biotechnol.

[CR4] Carvalho SMP, Vasconcelos MW (2013). Producing more with less: strategies and novel technologies for plant-based food biofortification. Food Res Int.

[CR5] Challis GL, Ravel J (2000). Coelichelin, a new peptide siderophore encoded by the Streptomyces coelicolor genome: structure prediction from the sequence of its non-ribosomal peptide synthetase. FEMS Microbiol Lett.

[CR6] Dodd NK, Pushpamma P (1980). Effects of locate and varieties on protein, amino acids and mineral contents of chick pea. Indian J Agric Sci.

[CR7] Fan MS, Zhao FJ, Fairweather-Tait SJ, Poulton PR, Dunham SJ, McGrath SP (2008). Evidence of decreasing mineral density in wheat grain over the last 160 years. J Trace Elem Med Biol.

[CR8] FDA (2010) Nutritional Labeling and Education Act (NLEA) Requirements (8/94-2/95), revised by 2010. Food and Drug Administration, USA

[CR9] Fiedler HP, Krastel P, Müller J, Gebhardt K, Zeeck A (2001). Enterobactin: the characteristic catecholate siderophore of Enterobacteriaceae is produced by *Streptomyces* species. FEMS Microbiol Lett.

[CR10] Gadd GM (2010). Metals, minerals and microbes: geomicrobiology and bioremediation. Microbiology.

[CR11] Gopalakrishnan S, Pande S, Sharma M, Humayun P, Kiran BK, Sandeep D, Vidya MS, Deepthi K, Rupela O (2011). Evaluation of actinomycete isolates obtained from herbal vermicompost for biological control of *Fusarium* wilt of chickpea. Crop Prot.

[CR12] Gopalakrishnan S, Vadlamudi S, Apparla S, Bandikinda P, Vijayabharathi R, Bhimineni RK, Rupela O (2013). Evaluation of *Streptomyces* spp. for their plant growth-promotion traits in rice. Can J Microbiol.

[CR13] Gopalakrishnan S, Vadlamudi S, Bandikinda P, Sathya A, Vijayabharathi R, Rupela O, Kudapa H, Katta K, Varshney RK (2014). Evaluation of *Streptomyces* strains isolated from herbal vermicompost for their plant growth-promotion traits in rice. Microbiol Res.

[CR14] Gopalakrishnan S, Vadlamudi S, Alekhya G, Prakash B, Kudapa H, Varshney RK (2015). Evaluation of broad-spectrum *Streptomyces* sp. for plant growth-promotion traits in chickpea. Philipp Agric Sci.

[CR15] Gopalakrishnan S, Vadlamudi S, Alekhya G, Prakash B, Kudapa H, Varshney RK (2015). Evaluation of *Streptomyces* sp. obtained from herbal vermicompost for broad spectrum of plant growth-promoting activities in chickpea. Org Agric.

[CR16] Gopalakrishnan S, Vadlamudi S, Alekhya G, Prakash B, Kudapa H, Rathore A, Varshney RK (2015). The extent of grain yield and plant growth enhancement by plant growth-promoting broad-spectrum *Streptomyces* sp. in chickpea. SpringerPlus.

[CR17] Graham RD, Welch RM, Saunders DA, Bouis HE, Bonierbale M, de Haan S, Burgos G, Thiele G, Liria R, Meisner CA, Beebe SE, Potts MJ, Kadian M, Hobbs PR, Gupta RK, Twomlow S (2007). Nutritious subsistence food systems. Adv Agron.

[CR18] Gupta AP (2005). Micronutrient status and fertilizer use scenario in India. J Trace Elem Med Biol.

[CR19] Hefnawy TH (2011). Effect of processing methods on nutritional composition and anti-nutritional factors in lentils (*Lens culinaris*). Ann Agric Sci.

[CR20] Ibáñez MV, Rincón F, Amaro M, Martínez B (1998). Intrinsic variability of mineral composition of chickpea (*Cicer arietinum*, L.). Food Chem.

[CR21] Imbert M, Bechet M, Blondeau R (1995). Comparison of the main siderophores produced by some species of *Streptomyces*. Curr Microbiol.

[CR22] Khalid S, Asghar HN, Akhtar MJ, Aslam A, Zahir ZA (2015). Biofortification of iron in chickpea by plant growth promoting rhizobacteria. Pak J Bot.

[CR23] Lee J, Postmaster A, Soon HP, Keast D, Carson KC (2012). Siderophore production by actinomycetes isolates from two soil sites in Western Australia. Biometals.

[CR24] Ma Y, Prasad MNV, Rajkumar M, Freitas H (2011). Plant growth promoting rhizobacteria and endophytes accelerate phytoremediation of metalliferous soils. Biotechnol Adv.

[CR25] Prasanna R, Bidyarani N, Babu S, Hossain F, Shivay YS, Nain L (2015). Cyanobacterial inoculation elicits plant defense response and enhanced Zn mobilization in maize hybrids. Cogent Food Agric.

[CR26] Rana A, Saharan B, Nain L, Prasanna R, Shivay YS (2012). Enhancing micronutrient uptake and yield of wheat through bacterial PGPR consortia. Soil Sci Plant Nutr.

[CR27] Rana A, Joshi M, Prasanna R, Shivay YS, Nain L (2012). Biofortification of wheat through inoculation of plant growth promoting rhizobacteria and cyanobacteria. Eur J Soil Biol.

[CR28] Rana A, Kabi SR, Verma S, Adak A, Pal M, Shivay YS, Nain L (2015). Prospecting plant growth promoting bacteria and cyanobacteria as options for enrichment of macro-and micronutrients in grains in rice–wheat cropping sequence. Cogent Food Agric.

[CR29] Sessitsch A, Kuffner M, Kidd P, Vangronsveld J, Wenzel WW, Fallmann K, Puschenreiter M (2013). The role of plant-associated bacteria in the mobilization and phytoextraction of trace elements in contaminated soils. Soil Biol Biochem.

[CR30] Singh MV (2009). Micronutrient nutritional problems in soils of India and improvement for human and animal health. Indian J Fertil.

[CR31] Vidal-Valverde C, Sotomayor JFC, Fernandez CDM, Urbano G (1998). Nutrients and antinutritional factors in faba beans as affected by processing. Z Lebensm Unters F.

[CR32] von Grebmer K, Saltzman A, Birol E, Wiesmann D, Prasai N, Yin S, Yohannes Y, Menon P, Thompson J, Sonntag A (2014) Global Hunger Index: The challenge of hidden hunger. Welthungerhilfe, International Food Policy Research Institute, and Concern Worldwide, Bonn, Washington DC, Dublin

[CR33] Welch RM, Singh K, Mori S, Welch RM (2001). Micronutrients, agriculture and nutrition; Linkages for improved health and wellbeing. Perspectives on the micronutrient nutrition of crops.

[CR34] White PJ, Broadley MR (2009). Biofortification of crops with seven mineral elements often lacking in human diets—iron, zinc, copper, calcium, magnesium, selenium and iodine. New Phytol.

[CR35] WHO (2002) The world health report 2002. World Health Organization, Geneva. http://www.who.int/whr/2002/. Accessed 31 Oct 15

[CR36] WHO (2004) Vitamin and mineral requirements in human nutrition. World Health Organization, Geneva. http://apps.who.int//iris/bitstream/10665/42716/1/9241546123.pdf. Accessed 31 Oct 15

